# Low frequency repetitive transcranial magnetic stimulation to the right dorsolateral prefrontal cortex engages thalamus, striatum, and the default mode network

**DOI:** 10.3389/fnins.2022.997259

**Published:** 2022-09-30

**Authors:** Elisabeth de Castro Caparelli, Osama A. Abulseoud, Hong Gu, Tianye Zhai, Brooke Schleyer, Yihong Yang

**Affiliations:** ^1^Neuroimaging Research Branch, National Institute on Drug Abuse, National Institutes of Health, Baltimore, MD, United States; ^2^Department of Psychiatry and Psychology, Mayo Clinic, Phoenix, AZ, United States; ^3^Department of Psychology, College of Liberal Arts, Temple University, Philadelphia, PA, United States

**Keywords:** DLPFC, fMRI, low frequency, multimodality, TMS

## Abstract

The positive treatment outcomes of low frequency (LF) repetitive transcranial magnetic stimulation (rTMS) when applied over the right dorsolateral prefrontal cortex (DLPFC) in treatment-refractory depression has been verified. However, the mechanism of action behind these results have not been well-explored. In this work we used simultaneous functional magnetic resonance imaging (fMRI) during TMS to explore the effect of LF rTMS on brain activity when applied to the right [RDLPFC1 (MNI: 50, 30, 36)] and left DLPFC sites [LDLPFC1 (MNI: -50, 30, 36), LDLPFC2 (MNI: -41, 16, 54)]. Seventeen healthy adult volunteers participated in this study. To identify brain areas affected by rTMS, an independent component analysis and a general linear model were used. Our results showed an important laterality effect when contrasting rTMS over the left and right sites. Specifically, LF rTMS increased brain activity at the striatum, thalamus, and areas of the default mode network when applied to the right, but not to the contralateral left DLPFC. In contrast, no site differences were observed when evaluating the effect of LF rTMS over the two left sites. These findings demonstrate that LF rTMS to the right DLPFC was able to stimulate the cortico-striato-thalamo-cortical pathway, which is dysregulated in patients with major depressive disorder; therefore, possibly providing some neurobiological justification for the successful outcomes found thus far for LF rTMS in the treatment of depression.

## Introduction

The efficacy of repetitive transcranial magnetic stimulation (rTMS) in treating certain neuropsychiatric disorders, specifically treatment-refractory depression (TRD) has been well-documented (Anand and Hotson, 2002; Gaynes et al., 2014; Wei et al., 2017). The dorsolateral prefrontal cortex (DLPFC) has conventionally been the preferred stimulation site for treatment of TRD (George et al., 1995). The cortico-striato-thalamo-cortical (CSTC) loop is a key network involved in emotional regulation (Peters et al., 2016) through heavy dopaminergic projections at individual nodes (Gerfen, 2000; Sanchez-Gonzalez et al., 2005). Major depression is associated with a state of reduced dopaminergic transmission along the CSTC loop (Dunlop and Nemeroff, 2007; Grace, 2016) and it is hypothesized that TMS over the DLPFC stimulates the CSTC pathway, improving the dopamine state (Strafella et al., 2001), therefore normalizing some affective and cognitive functions (Nitschke et al., 2004; Grimm et al., 2008; Goldstein and Volkow, 2011; da Silva et al., 2013).

In general, depression treatment protocols employ high frequency (HF—10 to 20 Hz) rTMS to the left DLPFC (Polley et al., 2011; McClintock et al., 2018). While the efficacy of this approach has been verified (George et al., 1995, 1997) and its mechanism explored (Strafella et al., 2001; Cho and Strafella, 2009; Caparelli et al., 2022), more recently it has been demonstrated that low frequency (LF) rTMS, which refers to a stimulation rate of ≤1 Hz (Wassermann, 1998), to the right DLPFC is as effective as HF rTMS to the left DLPFC in attenuating the severity of TRD (Chen et al., 2013; Garnaat et al., 2018). However, whether LF TMS activates the CSTC network, or other key limbic structures remains largely unknown.

In this study we aimed to investigate the differences in the mechanism of action for LF rTMS when applied to the right DLPFC and to the left DLPFC, since, clinically, the left DLPFC is the most commonly used stimulation target followed by the right DLPFC. Following the approach of our previous work (Caparelli et al., 2022), where the effect of HF rTMS was evaluated on three different DLPFC sites, we evaluated LF rTMS for the same three sites, two sites on the left: (1) Montreal Neurological Institute (MNI) coordinates = -50, 30, 36 [chosen because it has previously shown to have promising treatment results for cocaine addiction, such as, reduction of craving and cocaine use, as well as improvement of their depressive symptoms (Terraneo et al., 2016)], (2) MNI coordinates = –41, 16, 54 [chosen as a standardized metric, the “5 cm rule” (Fox et al., 2012)], and one on the right: MNI coordinates = 50, 30, 36 (the contralateral side of site #1), since the functional asymmetric aspect of the DLPFC has been previously reported (Chen et al., 2018). Here we aspire to verify the top-down mechanism of LF (0.4 Hz) rTMS on these three different DLPFC sites and to understand the site and laterality differences of LF rTMS.

## Materials and methods

The current study utilized LF (0.4 Hz) rTMS targeting the DLPFC, combined with the simultaneous fMRI recording. The study consisted of four study visits for each of the participants.

### Participants

Seventeen healthy adult volunteers (nine males, eight females, age: 37.1 ± 11.2 years old) participated in the study, among which fifteen were completers (participants who completed all four study visits) and two non-completers [one lost contact, and the other withdrew from the study (Caparelli et al., 2022)]. Participants with any neuropsychiatric disorder and/or any contraindication to fMRI scanning or rTMS administration were excluded (Caparelli et al., 2022). The study was approved by the Institutional Review Board panel of the National Institute on Drug Abuse and written informed consent was obtained from each participant.

### Experiment design and data acquisition

As previously described (Caparelli et al., 2022), this study was composed of four study visits (days). On the first day the three stimulation sites, LDLPFC1 (MNI: -50, 30, 36), LDLPFC2 (MNI: -41, 16, 54) and RDLPFC1 (MNI: 50, 30, 36) were localized and the resting motor threshold (rMT) was determined outside the MRI scanner. On the following three study days the simultaneous TMS-fMRI sessions (one stimulation site per day) were carried out using a TMS intensity of 100% of the rMT. The LF (0.4 Hz) rTMS sessions were carried out in the morning, and the HF (10 Hz) rTMS sessions (Caparelli et al., 2022) were acquired in the afternoon, with a minimum of 1.5 h between sessions.

#### Transcranial magnetic stimulation

The MagVenture system (MagPro X100 with MagOption stimulator, C-B60 coil, MRi-B91 Air Cooled coil–MagVenture Inc., Alpharetta, GA, USA) and the TMS Neuronavigation system (Brainsight–Rogue Resolutions Ltd, Montreal, QC, Canada) were used in this study (Caparelli et al., 2022). The neuronavigation system was used only for the rMT determination and localization of the stimulation sites. The identified stimulation sites were marked on the participant’s cap to guide the application of TMS stimulation on the following concurrent TMS-fMRI experiment days. For each TMS-fMRI session, the TMS coil was centered over the indicator for the stimulation site assigned for that day, with the order of the stimulation sites varied among subjects. Foam pads were used to minimize head motions (Caparelli et al., 2022).

#### Magnetic resonance imaging

**FIGURE 1:**
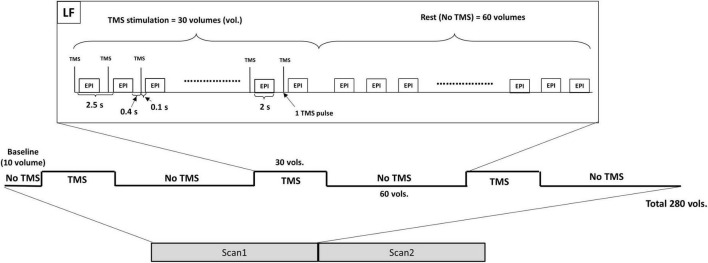
The TMS-fMRI session is composed of two fMRI scans. Each scan starts with a baseline followed by three stimulation blocks alternated by three resting blocks. During the stimulation block, each TMS pulse is applied 100 ms before the next EPI acquisition (400 ms after the previous EPI acquisition). TMS, transcranial magnetic stimulation; LF, low frequency; EPI, echo-planar imaging.

A 3 Tesla Prisma-fit Siemens scanner and a transmit-receive (Tx/Rx) single-channel birdcage head radio-frequency coil were used to acquire the fMRI data using a gradient-echo echo-planar imaging sequence (EPI–TE/TR of 27/2,500 ms, TR delay of 500 ms, spatial resolution of 3.4 × 3.4 × 4.4 mm^3^, 36 slices per volume, 280 volumes, axial orientation, flip angle of 78°) and the T1-weighted magnetization-prepared rapid gradient-echo (MPRAGE) images (Caparelli et al., 2022). Same as our previous work (Caparelli et al., 2022), each TMS-fMRI session was composed of two fMRI scans and each scan started with 10 baseline volumes, followed by six alternating blocks, three blocks of stimulation “ON” (30 volumes with TMS on) and three blocks of stimulation “OFF” (60 volumes with TMS off). During the stimulation block, the TMS pulses were applied in an interleaved fashion with the fMRI acquisition, and for every TR, the TMS pulse was applied 100 ms before the fMRI volume acquisition (Figure 1). Thus, the rTMS was applied at frequency of 0.4 Hz, i.e., one pulse every 2.5 s.

### Data analysis

Two approaches were considered for the analysis of the fMRI data, the Independent Component Analysis (ICA) and the General Linear Model (GLM) analysis. The analytical methods for both approaches have been detailed in our previous work (Caparelli et al., 2022), and summarized below. All analyses were carried out using FSL (the FMRIB Software Library, Oxford, UK) (Jenkinson et al., 2012), AFNI (Cox, 1996), or RStudio (RStudio Team, 2020).

#### Independent component analysis approach

##### Preprocessing, independent component analysis, and spatial regression

**FIGURE 2:**
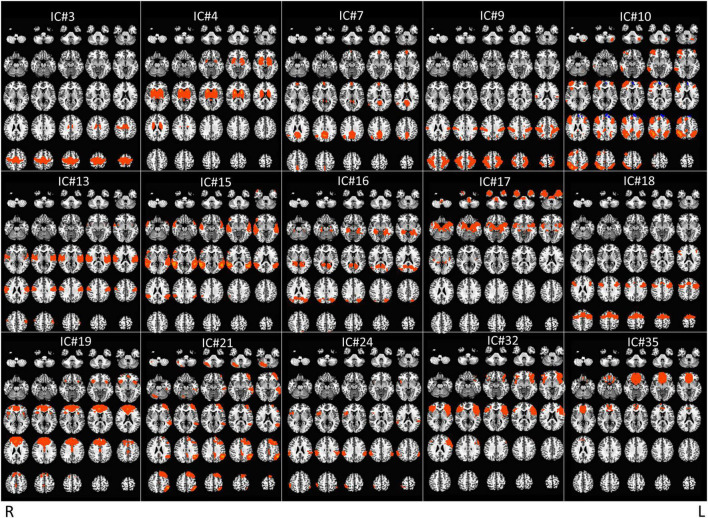
Display of the 15 meaningful ICs from group independent component analysis (ICA). Radiological convention.

The raw fMRI data were preprocessed (slice-timing and motion correction, spatial normalization to the MNI space, spatial smoothing, detrending, scaling); bad fMRI scans [with excessive motion (more than 30% of the volumes with motion above 0.3 mm of Euclidean distance) and/or any imaging artifact] were discarded whenever they could not be repeated (for final data set, see Table 1). Preprocessed data was then fed into the group ICA, which was carried out using MELODIC in FSL with the component number set at 40. Next, fifteen Independent Components (ICs) were chosen (Figure 2 and Table 2), after excluding some meaningless ICs [e.g., components showing motion artifacts, scattered small clusters across the brain, cerebral spinal fluid (CSF), white matter (WM), or components showing only either occipital or cerebellum which is considered to be minimally affected by TMS delivered over the DLPFC (Vink et al., 2018)—Supplementary Figure 2]. Following, a spatial regression analysis was conducted to extract the time courses for each IC, participant, scan, and stimulation site. The time courses of the six motion parameters and the average time courses extracted from the CSF and WM regions were regressed out from the IC time courses as nuisance regressors (Caparelli et al., 2022).

**TABLE 1:** Number of available data sets per functional magnetic resonance imaging (fMRI) scan and stimulation site.

	LF-LDLPFC1	LF-RDLPFC1	LF-LDLPFC2
Scan 1	10	12	13
Scan 2	12	13	13

LF, low frequency; LDLPFC1, left dorsolateral prefrontal cortex #1; LDLPFC2, left dorsolateral prefrontal cortex #2; RDLPFC1, right dorsolateral prefrontal cortex #1.

**TABLE 2:** Brain areas for the 15 independent components (ICs) presented in Figure 2.

IC #	Brain areas
3	Paracentral lobule, Medial frontal gyrus
4	Striatum, thalamus
7	DMN: superior medial gyrus, PCC, angular gyrus
9	DAN: superior and inferior parietal lobe, postcentral gyrus
10	Right ECN: right middle frontal gyrus, right and left inferior parietal lobe
13	Posterior insula, superior and transverse temporal gyri, lateral sulcus
15	Middle and superior temporal gyri, inferior parietal lobe
16	Amygdala, hippocampus, occipital, angular gyrus
17	Amygdala, hippocampus, temporal pole
18	SMA, left and right precentral gyri
19	Rostral ACC, ventromedial PFC, caudate
21	Left ECN: left middle frontal gyrus, left inferior parietal lobe
24	Right insula, left and right supramarginal gyri
32	Anterior insula, inferior frontal gyrus
35	Medial OFC and sgACC

IC, independent component; DMN, default mode network; PCC, posterior cingulate cortex; DAN, dorsal attention network; ECN, executive control network; SMA, supplementary motor area; ACC, anterior cingulate cortex; OFC, orbitofrontal cortex; sgACC, subgenual ACC.

##### Deconvolution

The IC time courses, for each IC, scan and stimulation site, were averaged across participants. Next, for each averaged IC time course, the hemodynamic response function (HRF) of the rTMS with duration of one stimulation-rest epoch [one stimulation block (TMS “ON”–30 images) + one resting block (TMS “OFF”–60 images)] was estimated through a linear regression with TENT function, which extracts the impulse response function for the averaged stimulation-rest epochs from the data. Following, the estimated HRF was smoothed and concatenated three times along with 10 time points of zeros values at the beginning of the vector, to generate the TMS response vector (TRV) for each IC, scan and stimulation site. Then, the TRVs, for each IC and stimulation site, were averaged across scans and correlated with the TMS paradigm (block design vector), just to determine the TRVs orientation. Finally, the signs of TRVs (from scan 1 and scan 2) were flipped only if correlation value was negative (Caparelli et al., 2022).

##### Correlation and *T*-tests

For each IC and stimulation site, the final TRV derived from one scan was correlated with the IC time course from the other scan for each participant [i.e., TRV (scan 1) correlated with IC time course (scan 2) and TRV (scan 2) correlated with IC time course (scan 1)]; finally, for each participant, the two correlation values were averaged and transformed to z-values through Fisher r-to-z-transformation.

Statistical analyses were carried out to evaluate the significant results within and between sites. One-sample *T*-tests were conducted for each IC and stimulation site, and the results were Bonferroni corrected for the 15 ICs. Subsequently, the site (LDLPFC1 vs. LDLPFC2) and laterality (LDLPFC1 vs. RDLPFC1) differences were evaluated using two-sample *T*-tests that only included ICs with significant results from the one-sample *T*-tests for at least one of the sites (six ICs were included for site differences and eight for laterality differences). The results were then Bonferroni corrected based on the respective numbers of ICs.

#### General linear model analysis

For comparison, a traditional GLM analysis was performed on the preprocessed fMRI data (slice-timing and motion correction, image spatial normalization to the MNI space, spatial smoothing and scaling) using the estimated time course constructed by convolving the block-design paradigm (TMS “ON” contrasting with TMS “OFF”) with the conventional HRF. Head motion parameters and average WM and CSF time courses were used as nuisance regressors in the first-level analysis, and volumes with excessive motion were censored out. For statistical analyses, one sample *T*-tests were conducted for each stimulation site, and two-sample *T*-tests were carried out to evaluate site and laterality differences.

## Results

### Independent component analysis approach

Results from the one sample *T*-test (Table 3) revealed a significant positive TMS effect at the posterior insula, superior and transverse temporal gyri, lateral sulcus (IC#13), and at the middle and superior temporal gyri and the inferior parietal lobe (IC#15) for all stimulation sites. A significant positive effect was also observed at the striatum, and thalamus (IC#4); and at the right insula and left and right supramarginal gyri (IC#24) when TMS was applied over the LDLPFC2 and RDLPFC1.

**TABLE 3:** One-sample *T*-test results.

	LF-LDLPFC1			LF-LDLPFC2			LF-RDLPFC1		
			
IC#	T-score	*P*-value	*p*_corr_	T-score	*P*-value	*p*_corr_	T-score	*P*-value	*p*_corr_
3	1.51	0.1601	ns	-1.68	0.1178	ns	0.29	0.7758	ns
4	1.72	0.1139	ns	5.25	0.0002	0.0031	5.44	0.0002	0.0023
7	0.03	0.9728	ns	1.17	0.2655	ns	7.19	1.10E-05	0.0002
9	0.49	0.6353	ns	2.11	0.0564	ns	-3.54	0.0041	ns
10	0.80	0.4406	ns	-0.15	0.8852	ns	-3.12	0.0089	ns
13	4.96	0.0004	0.0064	3.66	0.0033	0.0488	8.54	1.91E-06	2.87E-05
15	3.90	0.0025	0.0375	5.52	0.0001	0.0020	7.10	1.25E-05	0.0002
16	-6.77	3.06E-05	0.0005	-2.08	0.0597	ns	-3.20	0.0076	ns
17	-4.62	0.0007	0.0111	-1.50	0.1600	ns	-1.65	0.1243	ns
18	-0.41	0.6894	ns	0.31	0.7608	ns	-5.99	0.0001	0.0009
19	1.52	0.1569	ns	1.53	0.1527	ns	3.12	0.0088	ns
21	2.58	0.0257	ns	2.00	0.0689	ns	2.14	0.0540	ns
24	3.73	0.0033	ns	4.15	0.0014	0.0203	6.09	0.0001	0.0008
32	-0.60	0.5617	ns	1.30	0.2166	ns	1.37	0.1951	ns
35	-2.23	0.0474	ns	-1.83	0.0929	ns	0.48	0.6397	ns

*p*_corr_: Bonferroni corrected *p*-value for the 15 meaningful components (significant result, *p*_corr_ < 0.05). LF, low frequency; LDLPFC1, left dorsolateral prefrontal cortex #1; LDLPFC2, left dorsolateral prefrontal cortex #2; RDLPFC1, right dorsolateral prefrontal cortex #1; IC, independent component; *p*-value, uncorrected *p*-value; ns, not significant after Bonferroni correction.

An evident negative TMS effect was observed at the amygdala, hippocampus, occipital lobe, angular gyrus (IC# 16), and at the amygdala, hippocampus, and temporal pole (IC#17) when TMS was applied over the LDLPFC1. Negative TMS effects were also observed at the supplementary motor area (SMA), and the left and right precentral gyri (IC#18) when TMS was applied over the RDLPFC1. Significant positive TMS effect was observed at DMN (IC#7) only when TMS was applied over the RDLPFC1.

**FIGURE 3:**
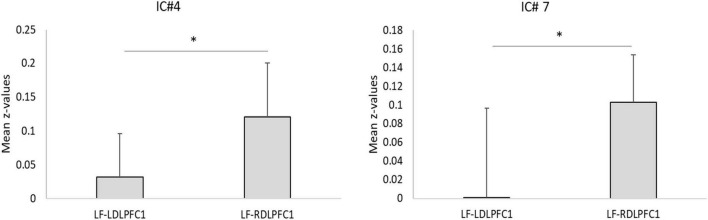
Laterality test. Plots for the mean correlation values (z-values) for ICs with significant laterality effects. *Two-sample *t*-test significant after Bonferroni correction as shown on Table 4 (*p_corr_ < 0.05). Error bars: standard deviation. LF, low frequency; LDLPFC1, left dorsolateral prefrontal cortex #1; RDLPFC1, right dorsolateral prefrontal cortex #1.

Results from the two-sample *T*-tests (Table 4 and Figure 3) showed no site difference when contrasting the LDLPFC1 stimulation affected networks with the LDLPFC2 stimulation affected networks. However, laterality differences were observed at the DMN (IC#7) and at the striatum and thalamus (IC#4) when contrasting the TMS effect over the left and right DLPFC1.

**TABLE 4:** Two-sample *T*-test results for site (LF-LDLPFC1—LF-LDLPFC2) and laterality (LF-LDLPFC1—LF-RDLPFC1) differences.

IC#	T-score	*P*-value	*p*_corr_
** LF-LDLPFC1—LF-LDLPFC2**			
4	–2.60	0.02	ns
13	1.79	0.09	ns
15	–0.93	0.36	ns
16	–2.06	0.05	ns
17	–1.83	0.08	ns
24	–0.97	0.34	ns
** LF-LDLPFC1—LF-RDLPFC1**			
4	–3.06	0.0055	0.044
7	–3.27	0.005	0.04
13	0.30	0.77	ns
15	–1.69	0.11	ns
16	–0.03	0.98	ns
17	–3.04	0.01	ns
18	2.74	0.01	ns
24	–2.60	0.02	ns

*p*_corr_: Bonferroni corrected *p*-value for the number of two-sample *T*-test performed on the meaningful components that showed significant one-sample *t*-test results (those pairs in which at least one of the conditions was significant after Bonferroni correction). *p*-value, uncorrected *p*-value; ns, not significant after Bonferroni correction; threshold of significance: *p*_corr_ < 0.05. LF, low frequency; LDLPFC1, left dorsolateral prefrontal cortex #1; LDLPFC2, left dorsolateral prefrontal cortex #2; RDLPFC1, right dorsolateral prefrontal cortex #1; IC, independent component.

### General linear model analysis

**FIGURE 4:**
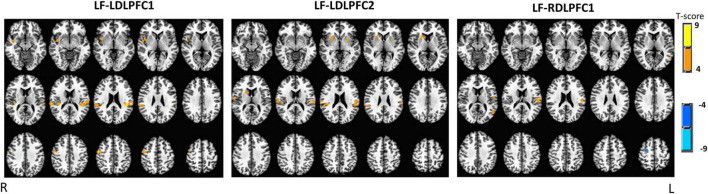
Results of general linear model (GLM) based analysis, one sample *T*-test for each stimulation site. Significance: uncorrected *p*-value (*p* = 0.001), corrected *p*-value (*p*_corr_ < 0.05). Radiological convention. LDLPFC1, left dorsolateral prefrontal cortex #1; RDLPFC1, right dorsolateral prefrontal cortex #1; LDLPFC2, left dorsolateral prefrontal cortex #2; LF, Low frequency; R, right; L, left.

Results for the traditional GLM-based analysis are shown on Figure 4. It revealed significant activation at the left and the right superior temporal gyri when TMS was applied over both left DLPFC sites (LDLPFC1 and LDLPFC2). Additionally, significant positive activation was observed at the left superior and middle temporal gyri when TMS was applied over the right DLPFC site (RDLPFC1).

However, positive activation at the right insula and precentral gyrus was observed only when TMS was applied over the LDLPFC1, and activation at right putamen and caudate was observed only when it was applied over the LDLPFC2. Conversely, significant de-activation was observed at the right superior frontal gyrus when TMS was applied over the RDLPFC1.

Neither site differences, contrasting between the two left DLPFC sites (LDLPFC1—LDLPFC2), nor laterality differences, contrasting between the left and right DLPFC1 sites (LDLPFC1—RDLPFC1), were observed when using the GLM analysis method.

## Discussion

The results of this study reveal an important mechanistic aspect of LF rTMS over the right DLPFC through activating the striatum and thalamus (CSTC loop) and the DMN. Our results suggest a potential mechanism of action of LF rTMS to the right DLPFC that has not been explored before, which may explain the positive outcome of LF rTMS when treating depression.

### Low frequency repetitive transcranial magnetic stimulation over the right dorsolateral prefrontal cortex—treatment

The role of CSTC dysregulation in depression has been well-studied (Peters et al., 2016) and a recent meta-analysis revealed that an antidepressant response from rTMS could be predicted by baseline DMN network connectivity (Long et al., 2020). In regard to the effect of depression on relevant network connectivity, there is evidence for enhanced connectivity (Rubart et al., 2018) associated with increased stimulation-induced activation (Sheline et al., 2009) within the DMN. These changes within the DMN correlated with depression severity and feelings of hopelessness in patients with TRD (Grimm et al., 2009); besides, one study showed TMS successfully induced anticorrelated connectivity between the DLPFC and medial prefrontal DMN nodes (Liston et al., 2014).

The efficacy of LF rTMS applied to the right DLPFC has also been documented in patients with TRD. In a meta-analysis of eight studies (*n* = 263), 34.6% of patients receiving active stimulation were classified as remitters (resolution of symptoms) compared to 9.7% receiving sham stimulation (*P* < 0.0001) (Berlim et al., 2013). Similarly, in another study, LF (1 Hz) rTMS applied over the right DLPFC produced a clinically relevant reduction in depressive symptomatology for TRD patients comparable to venlafaxine ER, an oral antidepressant medication, in a 4-week, double-blind study (rTMS with placebo vs. venlafaxine ER with sham rTMS) (Bares et al., 2009).

In contrast, LF (1 Hz) stimulation [contrasted with high (17 Hz) frequency and sham stimulations] targeting the left DLPFC in TRD patients (*n* = 72) did not show significant differences in depression severity, when compared active vs. sham rTMS (Miniussi et al., 2005). However, in another study LF TMS (1 Hz), applied over the right DLPFC, had the same effect as HF TMS (20 Hz), applied over the left DLPFC (Isenberg et al., 2005). Moreover, both LF TMS to the right DLPFC and HF TMS to the left DLPFC reduced depression severity, as assessed by the Beck Depression Inventory (BDI-II) (Beck et al., 1961) and the Clinical Global Impression (Busner and Targum, 2007) of Change (CGIC) scores, suggesting that rTMS applied at LF over the right frontal cortex appears to be as effective for treatment of refractory depression as HF rTMS over the left frontal cortex.

Additionally, 1 Hz rTMS over the right DLPFC decreased total scores on the Hamilton Depression Rating Scale (HAM-D) (Hamilton, 1960) and the Hamilton Anxiety Rating Scale (HAM-A) (Hamilton, 1959), and was effective in 42.9% of TRD subjects in the sample (Pallanti et al., 2012). Furthermore, 1 Hz rTMS over the right DLPFC improved health-related quality of life in unipolar and bipolar TRD patients (Dumas et al., 2014), and also improved generalized anxiety disorder symptoms, such as excessive worry and depressive symptoms (Diefenbach et al., 2016).

Finally, a meta-analysis has shown that LF (≤1 Hz) rTMS applied to the right DLPFC was as effective as HF (10–20 Hz) rTMS applied to the left DLPFC, on treating major depressive disorder (MDD), however LF right-sided rTMS produced fewer side effects and had less risk for seizures; suggesting that its clinical applicability is more promising and should be explored further (Chen et al., 2013). Taken together, the efficacy of low frequency stimulation at the right DLPFC is evident and comparable to the standard high frequency stimulation at the left DLPFC. However, the neurobiological underpinnings of LF- right DLPFC TMS remain under investigation.

### Implications of main findings

Our current study provides mechanistic understandings underlying the effectiveness of LF rTMS over the right DLPFC. The increased activity at the striatum and thalamus after LF rTMS to the right DLPFC, observed in this current work, was also observed in previous studies, after HF rTMS was applied to the left DLPFC (Strafella et al., 2001; Caparelli et al., 2022). These findings, in healthy volunteers, demonstrate the ability of TMS to stimulate the CSTC loop, either through LF to the right or the HF to left DLPFC, thus suggesting the modulation of the dysregulated dopamine reward circuitry in patients with MDD (Ko et al., 2008; Dunlop et al., 2016). It, therefore, justifies, at least in part, the significant efficacy of rTMS in attenuating symptom severity in patients with TRD. In addition, an increase in activity at the DMN was observed when LF rTMS was applied to the right DLPFC. Because DMN activity is considered a core component of pathological network dysfunction, since it compromises the ability for dynamic network change in response to changing demands in an otherwise healthy brain (Garnaat et al., 2018), our finding are in agreement with other studies that have reported that the positive outcome in treating depression through LF-TMS applied to the right DLPFC, is associated with the regulation of DMN activity by TMS (Sheline et al., 2009; Liston et al., 2014).

The observed increase in brain activity after LF rTMS to the right DLPFC is also in line with previous work, in which 1 Hz stimulation to the right DLPFC increased brain activation at the right DLPFC, during a decision-making gambling task, and improved anxiety, worry and depressive symptoms (Diefenbach et al., 2016). These, findings not only support the therapeutic effect of LF rTMS, but also demonstrate that LF rTMS does not always induce an inhibitory effect (Chen et al., 1997; Romero et al., 2002). In contrary, these results suggest that LF rTMS to the right DLPFC may have the effect of normalizing the imbalance of right and left prefrontal activity (Noda, 2021).

### Secondary findings

Although no site effect was observed when contrasting the results of LF rTMS over the two sites for the left DLPFC, some results from the one-sample *T*-test analysis may indicate specific LF rTMS effects for each stimulation site. For example, LF rTMS over the LDLPFC1 induced a decrease in activity at amygdala, hippocampus, occipital cortex, angular gyrus, and temporal pole (ICs #16 and #17). This finding is supported by previous work that showed that 1 Hz rTMS over the left PFC at 100% of the MT decreased regional cerebral blood flow (rCBF) at right prefrontal cortex, left medial temporal cortex, left basal ganglia, and left amygdala (Speer et al., 2000). On the other hand, the increased activity at insula (IC#24) observed when LF rTMS was applied to the RDLPFC1 and LDLPFC2, seems to contradict previous finding, where in a SPECT study 1 Hz rTMS over the right PFC, in TRD patients, showed significant decreases in rCBF at the in the PFC, OFC, sgACC, globus pallidus, thalamus, anterior and posterior insula, and midbrain in the right hemisphere (Kito et al., 2011). In addition, our finding shows a decrease in brain activity at the SMA and motor areas (IC#18) when LF rTMS was applied to the RDLPFC1, which opposes previous finding that have shown a decrease in the rMT after patients received 1 Hz rTMS over the right DLPFC (Pallanti et al., 2012). Several factors may contribute to these seemingly contradicting results, for example, those findings were reported in TRD patients, and ours are in healthy controls. In addition, the stimulation site on the SPECT study was at the PFC at large, without specifically targeting the DLPFC. Furthermore, it should be noted that most of the LF rTMS studies cited here used 1 Hz rTMS and ours used 0.4 Hz rTMS, which may introduce some divergences on the findings. Further research is needed to delineate the effect of LF (0.4 Hz) rTMS over the right DLPFC on regional brain activation and network functional connectivity in TRD patients.

### General linear model results

The GLM findings, although showing very few significant clusters, still reproduced the ICA findings in a shorter scale. For example, the activation observed at right putamen and caudate when rTMS was applied over the LDLPFC2 overlapped with the significant finding at IC#4, and the significant negative activation observed at the right superior frontal gyrus when rTMS was applied over the RDLPFC1 overlapped with the significant finding at IC#18. Overall, the complete overlap of the results between the two analysis methods strongly supports our findings.

### Non-transcranial magnetic stimulation related findings

We observed increased activity in the auditory cortex for all three sites (IC#13 and #15). This effect, although weaker, was also observed in the GLM findings (Figure 4 and Supplementary Figure 1). This activity at the auditory cortex is probably induced by the acoustic noise produced by the TMS coil, when delivering pulses, not necessarily related to magnetic stimulation. Although these results show that the TMS pulse was not masked and therefore likely created an anticipatory effect since the pulses came at predictable times, these effects were eliminated when contrasting each pair of stimulation sites (two sample *T*-test), showing that the effect of auditory stimulation and its anticipation were removed when the sites were contrasted.

### Limitations

The current study carries few limitations as described below.

(1) The lack of sham. The lack of sham is a limitation in this study, however, most of the non-magnetic stimulation sources that the sham stimulation intends to control, such as, TMS “tap” sensations and acoustic noise, were either mostly eliminated, when contrasting the brain activation from each pair of sites, or not significant, due to the study design. For example, the TMS acoustic noise, which mostly depend on the TMS frequency and intensity, were fixed within subjects. Consequently, the results for the IC# 15, which includes the auditory cortex, were not significant when contrasting two stimulation sites. The “tap” sensation, for the very low frequency (0.4 Hz), may have been subtle, since there were no major complaints about pain or discomfort during these low frequency sessions.
(2) Small sample size. Although, small sample size on simultaneous TMS-fMRI studies is not uncommon, and have proofed to find meaningful findings (Ruff et al., 2008; de Weijer et al., 2014; Hawco et al., 2017; Navarro de Lara et al., 2017; Dowdle et al., 2018; Vink et al., 2018; Luber et al., 2022), it still may represent a limitation on this study. Therefore, besides choosing an analysis streamline that minimize, as much as possible, the excessive use of the data, we focus our discussion on the results from ROI analysis defined by ICA, instead of the GLM (voxel-wise analysis) results, which provided very few significant activation clusters as shown on Figure 4. Consequently, while findings from this work should be considered preliminary, given the small to moderate sample size, some interesting hypotheses (as described above) were generated based on our current findings.

### General linear model vs. independent component analysis

Traditional GLM analysis on block-design fMRI data is performed by convolving the boxcar stimulus with the canonical HRF to construct the expected brain response function. However, the canonical HRF was estimated based on task-induced fMRI signal changes and typically assumed spatially invariance across the brain, which might not be suitable to analyze TMS-induced brain response. Therefore, the analysis framework, developed in our previous work (Caparelli et al., 2022), was employed here to estimate the brain responses induced by the TMS “ON” blocks in different brain networks. The brain responses were estimated assuming the brain as a time-invariant system by averaging the fMRI time series across the TMS ON-OFF periods (stimulus-rest epochs). More specifically, the HRF for TMS stimulation was estimated using the TENT function, which extracted the impulse response function for the averaged stimulus-rest epoch from the data.

Compared with traditional GLM analysis, the ICA-based analysis used in this study, which employs the data-driven estimated HRF, was more sensitive on detecting TMS-induced brain activity changes by estimating the TMS-induced brain responses directly from the data and not assuming fixed responses across the brain networks.

## Overall conclusion

In conclusion, here we showed that LF (0.4 Hz) rTMS to the right DLPFC has a similar top-down mechanism to, the previously reported, HF rTMS stimulation to the left DLPFC, which has shown to activate brain circuitry that are dysfunctional in MDD. This finding brings new insights into the mechanism behind LF rTMS to the right DLPFC and justifies its positive clinical outcomes. Finally, given the fewer side effects of LF rTMS when compared with HF rTMS, its clinical applicability is more promising.

## Data availability statement

The datasets presented in this article are not readily available because the availability of any human data will follow local institutional regulations, where data was generated. Requests to access the datasets should be directed to the corresponding author.

## Ethics statement

The studies involving human participants were reviewed and approved by Institutional Review Board panel of the National Institute on Drug Abuse. The patients/participants provided their written informed consent to participate in this study.

## Author contributions

EC designed the study, carried the experimental setup, participated in data collection, data analysis, and manuscript preparation. OA participated in data collection and provided relevant inputs for the introduction and discussion. HG provided input in data analysis and relevant inputs on the interpretation of the results. TZ provided input in the study design and data analysis and participated in the experimental setup and some data collection. BS participated in data collection. YY provided input in the study design and data analysis and provided the financial support. All authors contributed to manuscript revision and approved the submitted version.
