# Supplementary material for: Low frequency repetitive transcranial magnetic stimulation to the right dorsolateral prefrontal cortex engages thalamus, striatum, and the default mode network
Source: Front Neurosci. 2022 Sep 30;16:997259. doi: 10.3389/fnins.2022.997259 (PMC9565480; doi:10.3389/fnins.2022.997259)
Supplement: Supplementary file 1 [file Data_Sheet_1.PDF]

Supplementary figures

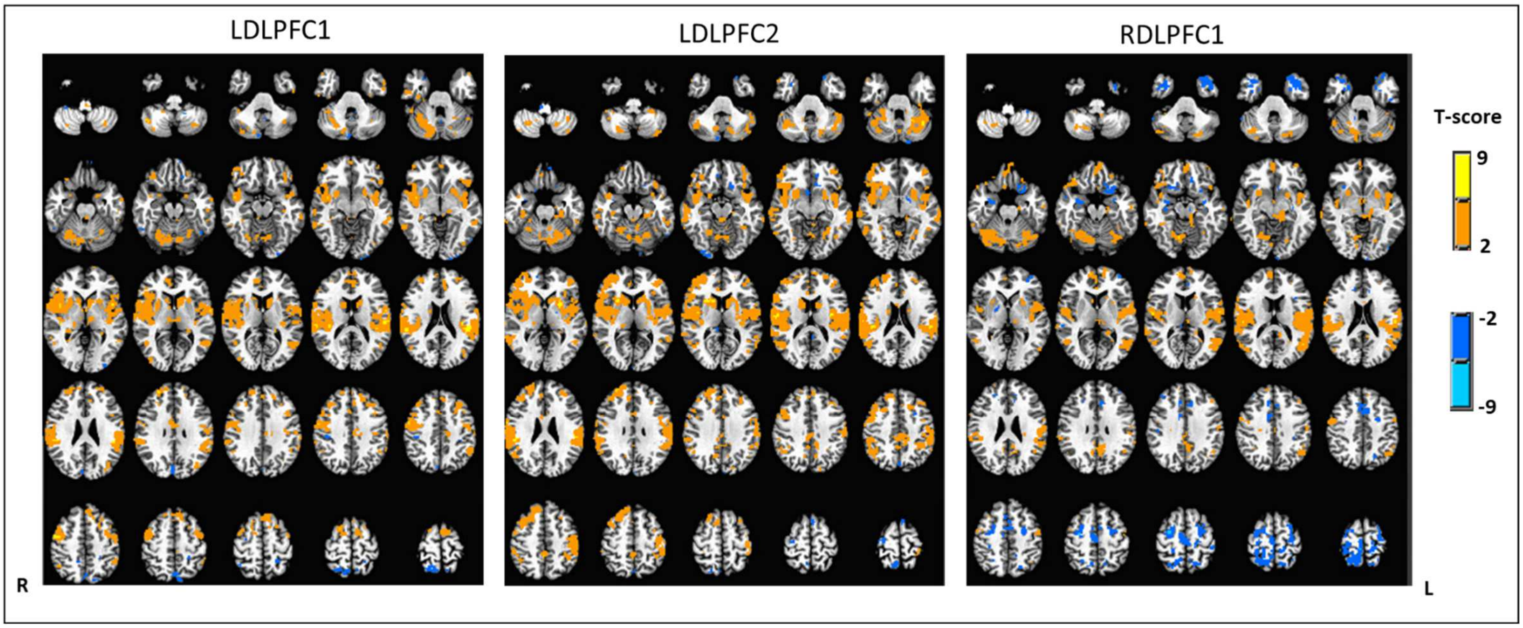

Figure S1: Results of GLM analysis, one sample T-test for each stimulation site. Significance: uncorrected *p*-value ($p = 0.05$). Radiological convention. Abbreviations: LDLPFC1: left dorsolateral prefrontal cortex #1, RDLPFC1: right dorsolateral prefrontal cortex #1, LDLPFC2: left dorsolateral prefrontal cortex #2, LF: Low frequency, R: right, L: left.

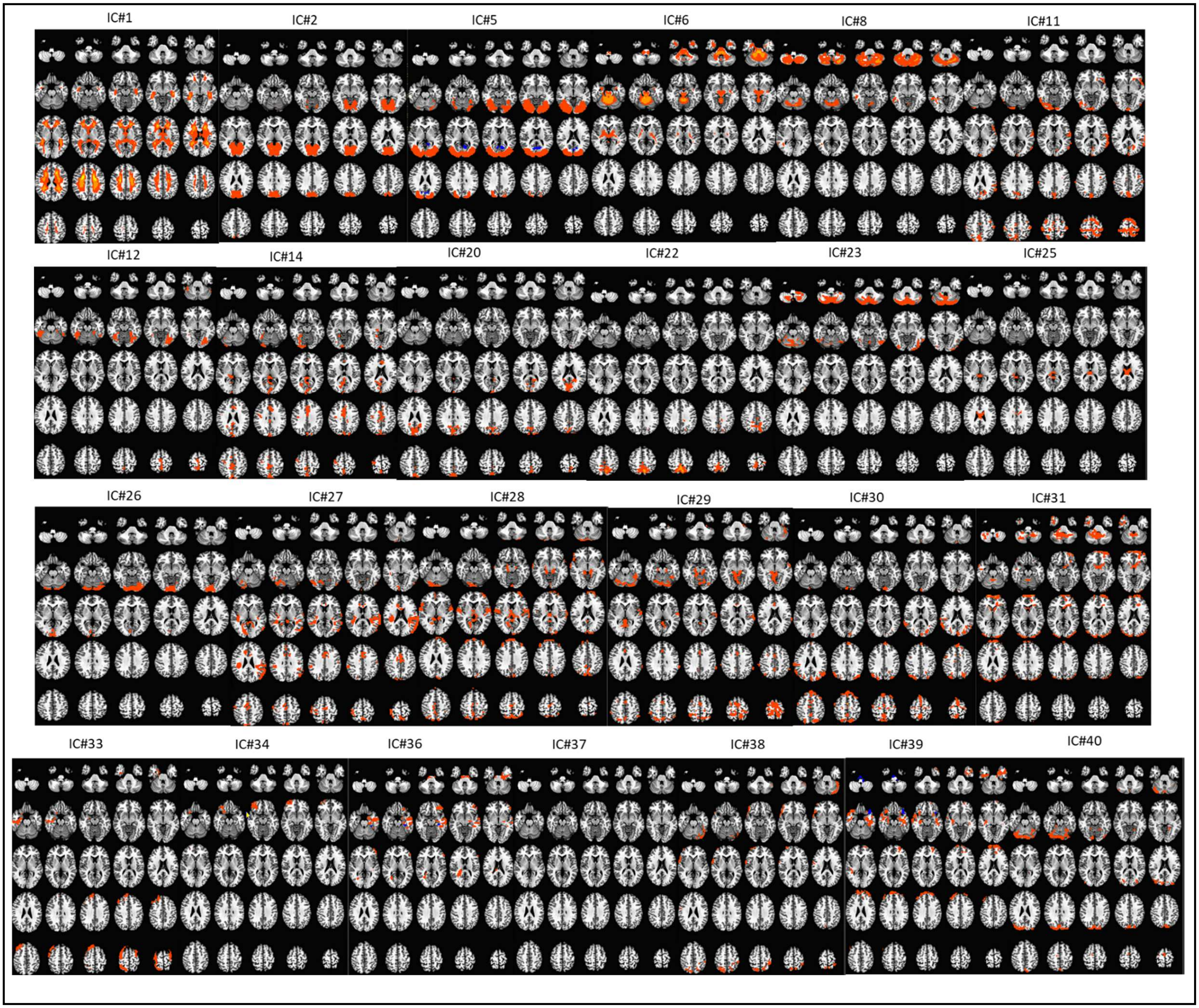

Figure S2: Excluded components from ICA analysis. WM (#1), CSF (#25), motion (#31, #39, #40), cerebellum (#6, #8, #23), occipital (#2, #5, #22, #26). All the remain are components that shows scattered small clusters across the brain. The figure was created with a threshold of 200 pixels per clusters to eliminate noise, on IC#37 no cluster survived the threshold.
